# Enhanced Production of Insulin-like Growth Factor I Protein in *Escherichia coli *by Optimization of Five Key Factors

**Published:** 2015

**Authors:** Javad Ranjbari, Valiollah Babaeipour, Hossein Vahidi, Hamidreza Moghimi, Mohammad Reza Mofid, Mohammad Mehdi Namvaran, Sevda Jafari

**Affiliations:** a *Department of Pharmaceutical Biotechnology, School of Pharmacy, Shahid Beheshti University of Medical Sciences, Tehran, Iran.*; b *Department of Bioscience and Biotechnology, Malek Ashtar University of Technology, Tehran, Iran.*; c *Department of Life Science Engineering, School of New Science and Technologies, University of Tehran, Iran.*; d *Department of Pharmaceutics, School of Pharmacy, Shahid Beheshti University of Medical Sciences, Tehran, Iran.*; e *Department of Biochemistry, School of Pharmacy, Isfahan University of Medical Sciences, Isfahan, Iran.*

**Keywords:** rhIGF-I, *E. coli*, Non- continuous fermentation, Taguchi design of experiments, Optimization

## Abstract

Human insulin-like growth factor I (hIGF-I) is a kind of growth factor with clinical signiﬁcance in medicine. Up to now, *E. coli* expression system has been widely used as a host to produce rhIGF-1 with high yields. Batch cultures as non-continuous fermentations were carried out to overproduce rhIGF-I in *E. coli*. The major objective of this study is over- production of recombinant human insulin-like growth factor I (rhIGF-I) through a developed process by recruiting effective factors in order to achieve the most recombinant protein. In this study we investigated the effect of culture medium, induction temperature and amount of inducer on cell growth and IGF-1 production. Taguchi design of experiments (DOE) method was used as the statistical method. Analysis of experimental data showed that maximum production of rhIGF-I was occurred in 32y culture medium at 32 °C and 0.05 Mm IPTG. Under this condition, 0.694 g/L of rhIGF-I was produced as the inclusion bodies. Following optimization of these three factors, we have also optimized the amount of glucose and induction time in 5 liter top bench bioreactor. Full factorial design of experiment method was used for these two factors as the statistical method. 10 g/L and OD_600_=5 were selected as the optimum point of Glucose amount and induction time, respectively. Finally, we reached to a concentration of 1.26 g/L rhIGF-1 at optimum condition.

## Introduction

Human insulin-like growth factor I (hIGF-I), also known as somatomedin C, is a protein encoded in humans by the IGF-I gene. It consists of 70 amino acids and three disulﬁde bonds, which mainly exist in human liver and serum (1). As the biological characterization and activity, IGF-I is considered as an attractive issue in pharmaceutical industry. It regulates cell growth and differentiation of various cell types, improves glomerular ﬁltration and renal plasma ﬂow (2, 3). It plays an important role in childhood growth and continues to have anabolic effects in adults. A synthetic analog of IGF-1, mecasermin is used for the treatment of growth failure (4). It has 50% similarity to insulin and can increase insulin sensitivity and decrease the amount of glucose levels (3, 5). Also, it is currently being developed as a therapeutic agent in cancer therapy; tissue reconstruction and insulin-resistant diabetes remedy (6). Therfore, we designed this study to optimize IGF-I production.

Recombinant human IGF-I has been produced via recombinant DNA technology using a variety of host systems. So far, a variety of expression systems have been established to produce hIGF-1, including *Escherichia coli*, yeast, cell-free system (7, 8), transgenic plants (9, 10). *E. coli* has some advantages rather than other hosts such as easy handling and culture, and high yields. These characterizations make it most popular as a host to produce IGF-1(11, 12). In this project we used origami (B/DE3) strain. Origami (B/DE3) has mutations in both thioredoxin reductase (trxB) and glutathione reductase (gor) genes which greatly enhance disulfide bond formation in the *E. coli* cytoplasm. Then, Origami (B/DE3) is recommended only for the expression of proteins that require disulfide bond formation for proper folding (13,14).

The goal of enhancement and optimizing the production of recombinant proteins is to produce higher amount of functional product per unit volume per unit time (15). For *E. coli*, or any other fermentation system, the level of intracellular accumulation of recombinant protein is dependent on ﬁnal cell density and the speciﬁc activity of the protein. Several methods have been developed for increasing recombinant protein production. These methods can be categorized as four strategies, namely, choice of culture media, mode of cultivation, strain development, and expression system control (16, 17). The present study intends to evaluate the effects of medium type, temperature of induction, amount of inducer, amount of Glucose and induction time on increasing the production of rh-IGF-1 in batch culture. Experimental design is the process of planning a study to meet the specified objectives. Proper planning of an experiment is critical with the purpose of ensuring that the correct data and right sample size are available to answer the questions as clearly and efficiently as possible. Among various statistical experimental designs, Taguchi experimental design offers distinct advantages by which many factors can be examined simultaneously and much quantitative information can be extracted with a few experimental trials also facilitates to identify the influence of individual factors, and establish the relationship between variables and operational conditions (18, 19). In this study, the settings of the process parameters were determined by using full factorial and Taguchi’s experimental design method. Orthogonal arrays of Taguchi, full factorial arrays, the signal-to-noise (S/N) ratio, the analysis of variance (ANOVA) and regression analyses are employed to find the optimal process parameter levels and to analyze the effect of these parameters on rhIGF-1 production (20, 21). 

## Experimental

*Strain and plasmid*

**Figure 1:**
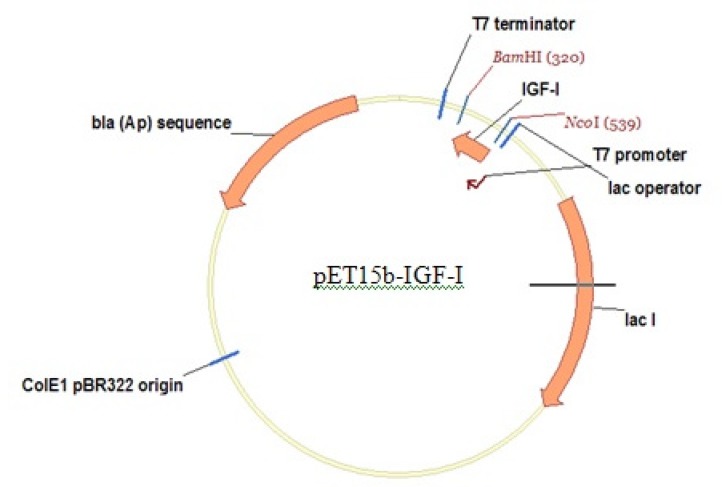
Expression vector: pET15b-rhIGF-1, ampicillin resistant with strong bacteriophage T7 promoter.

*E. coli* strain origami (B/DE3) (Novagen) was the chosen host. Origami (B/DE3) has mutations in both the thioredoxin reductase (trxB) and glutathione reductase (gor) genes, which greatly enhance disulfide bond formation in the *E. coli* cytoplasm. Then, Origami (B/DE3) is recommended only for the expression of proteins that require disulfide bond formation for proper folding. The Origami (B/DE3) strain is tetracycline resistant. PET15b plasmid (Novagen), an ampicillin resistant plasmid, was used as the vector in this work and synthetic IGF-1 gene (synthesis was performed by Gene Script Inc (USA)) was cloned in PET15b plasmid under control of strong bacteriophage T7 promoter (Figure 1). 

*Culture media*

Luria–Bertani (LB), TB and 32y culture media were used for optimization process:

LB (Merck Company) composition was: trypton 10 %(w/v), yeast extract 5 %(w/v), NaCl 10 %(w/v). 

TB (Merck Company) composition was: Peptone 1.2 %(w/v), yeast extract 2.4% (w/v), Glycerol 0.4 %(w/v), K_2_HPO_4_ 12.54 %( W/V), KH_2_PO_4_ 2.31 %( w/v).

32Y (Merck Company) composition was: peptone 0.8 %(w/v), yeast extract 3.2% (w/v), NaCl 0.58 (w/v) in Tris-HCl 10 mM (pH=7.6).

*Transformation and expression of rhIGF-1*

The expression vector pET15b-rhIGF-1 was transformed into *E. coli* strain origami (B/DE3). The transformed colonies were grown in 5 mL LB containing 100 μg/mL ampicillin and 100 μg/mL tetracycline at 37 °C. When OD_600_ reached 0.6–0.8, isopropyl-β-D-thiogalactopyranoside (IPTG) (Sigma-Aldrich) was added to a final concentration of 0.1 mM. The culture was incubated at 37 °C for 4 h. Then, expression of IGF-1 was analyzed through SDS-polyacrylamide gel electrophoresis (PAGE).

*Western blot analysis of rhIGF-1*

The expressed rhIGF-1 was also analyzed by Western blotting using anti-human IGF-1 antibody (Abcam-USA) for further identification. Briefly, 20-30 μg of total protein from cell lysate was loaded into the well of the SDS-PAGE gel 17.5%, along with molecular weight markers (Sigma). Next, gel was run for 1 to 2 hours at 100 V. Protein was transferred to nitrocellulose membrane (Bio-Rad). Membrane was blocked for 1 hour at room temperature using 5% blocking solution (PBS containing 5% milk). Then, Membrane was incubated with mouse anti-human IGF-1 monoclonal antibody in 5% blocking solution overnight at 4 °C. After washing with PBS for five times, the membrane was incubated with horse radish peroxidase conjugated secondary antibody in 5% blocking buffer for 1.5 h with shaking at room temperature. After washing with PBS for five times, the membrane was then visualized using an enhanced-chemiluminescence kit (FIVEphoton Biochemicals) by following the protocol as the manufacturer suggested.

*Experimental design*

Taguchi design of experiments (DOE) method and full factorial method were used as statistical methods. Our purpose in this study includes:

1. Selection of a suitable medium and optimization of the temperature of induction and the amount of inducer in shaking flask: three different levels were considered for each factor. Media including LB, TB and 32y were used for cultivation of recombinant *E. coli*. Three levels were also considered for temperature and the amount of inducer. In order to optimization L_9_ orthogonal array of the Taguchi tables was selected (Table. 1).

2. Selection of optimum amount of glucose and induction time in bioreactor (5 l bench top Mini Force bioreactor made in Switzerland): three levels were considered for each factor. Full factorial design of experiments (DOE) method was used as the statistical method (Table 2).

**Table 1:** Taguchi orthogonal table L9 for three-three level factors

Medium type	Inducer amount (mM)	Induction temperature (^o^C)	Number of experiment
LB	0.05	24	1
TB	0.1	24	2
32y	0.2	24	3
LB	0.1	28	4
TB	0.2	28	5
32y	0.05	28	6
LB	0.2	32	7
TB	0.1	32	8
32y	0.05	32	9

At first culture medium type, temperature of induction and amount of inducer were optimized in shaking flask. And then, the optimization of glucose and induction time was performed in a 5 L bench top bioreactor.

**Table 2:** Full factorial statistical analysis of two-three level factors

Number of experiment	Glucose amount	Induction time (OD_600_)
1	0 g/L	1
2	10 g/L	1
3	20 g/L	1
4	0 g/L	2.5
5	10 g/L	2.5
6	20 g/L	2.5
7	0 g/L	5
8	10 g/L	5
9	20 g/L	5

*Fermentation process*

*Shaking flask experiments*

Inocula were prepared by inoculating single colonies from a freshly spread plate into 10 mL of culture medium with 100 µg/mL Amp and 100 µg/mL tetracycline. The seed cultures were grown at 37 °C for about 8h on a shaker incubator at 120 rpm. The ﬁnal *A*_600_ value was typically about 1. Flask cultures (10 %( v: v)) in 250 mL ﬂasks were inoculated and shaken at 120 rpm; and prepare the temperature according to Taguchi table (Table 1) in a shaker incubator. Then, each flask was induced with IPTG at various concentrations according to Taguchi Table.

*Bioreactor experiments*

Cultures were grown in 32y medium. Antifoam (Sigma) was used. Each bioreactor initially contained 1800 mL medium and was inoculated with 200 mL of inoculums (freshly prepared as described below). The dissolved oxygen level was determined with a steam-sterilizable polarographic electrode and the culture pH was measured with a steam-sterilizable pH probe. Medium PH was controlled at pH=7 until induction by the automatic addition of HCL and NaOH. Cultures were induced with IPTG at various concentrations when biomass attained a level corresponding to *A*-value of 5.0. During all tests, the agitation speed and air ﬂow rate were adjusted to keep the dissolved oxygen level above 5% relative to saturation. Samples were taken at various intervals to measure cell concentration, rhIGF-1 level.

*Inoculum preparation*

Inoculum for bioreactor was obtained by two-stage cultivation. 250 mL ﬂask containing 20 mL of 32y medium was inoculated with a single colony from a freshly-spread plate and cultured for 8 h at 37 °C at 120 rpm. Then, a 1 L ﬂask containing 200 mL of 32y medium was inoculated with the culture from the 250 mL flask and incubated for 3 h at 37 °C at 120 rpm. Fermentation process was carried out in a 5 lit bench top bioreactor.

*Analytical procedures*

Cell growth was monitored by measuring culture turbidity and dry cell weight (DCW). Turbidity was determined by measuring the optical density (OD) at 600 nm. Samples were diluted with NaCl solution (9 g/L) to obtain an OD between 0.2 and 0.5. In order to determine DCW, 5 ml of broth culture was centrifuged at 4,000×g for 10 min, washed twice with deionized water, and dried at 105 °C to constant weight. Expressed recombinant protein was determined and quantiﬁed by SDS-PAGE, densitometry (Image J Software), and Bradford methods (22). Densitometry is the quantitative measurement of optical density in light-sensitive materials, such as photographic paper or photographic film, due to exposure to light. Optical density is a result of the darkness of a developed picture and can be expressed absolutely as the number of dark spots (*i.e*., silver grains in developed films) in a given area, but usually it is a relative value, expressed in a scale. Densitometry is particularly useful due to its sensitivity, accuracy and versatility, and it can be applied to proteins in gels or on membranes. In addition to being accurate, sensitive and reproducible, the technique is cost-effective, simple, and does not require a high degree of specialized training, yet provides technical advantages over other available tool (23). The rhIGF-1expression level was determined by SDS-PAGE on Polyacrylamide 17% (w/v). Gels were stained with Coomassie brilliant blue R250 and then quantiﬁed by gel densitometer. Bradford protein assay was used for the quantitative analysis of total protein.

## Results and Discussion

*Transformation and expression of rhIGF-1*

**Figure 2:**
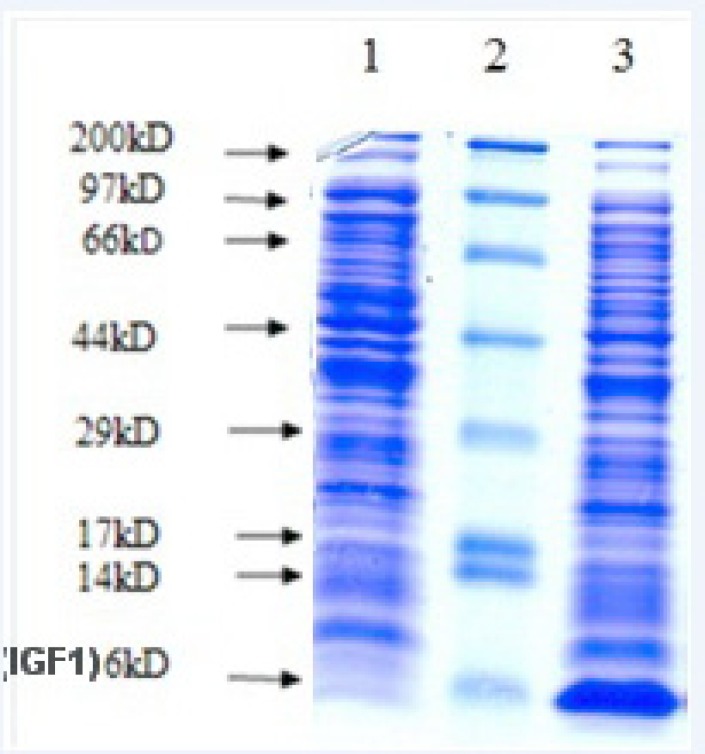
Expression of rhIGF-1 in batch fermentation in LB medium. Lane1: before induction. Lane 2: Marker, lane 3: after induction. Origami (B/DE3) ]pET15b-rhIGF-1[with 0.1 Mm IPTG

DNA sequence encoding human IGF-1 was cloned in pET15b vector to construct the recombinant plasmid pET15b-hIGF-1containing the exact human IGF-1 gene sequence confirmed by automated DNA sequencing. The expression plasmid was then transformed into *E. coli *strain Origami (B/DE3). In the presence of 0.1 mM IPTG, the expression of the protein was induced. After induction, transformed cells were analyzed for rhIGF-1 expression by SDS-PAGE (Figure 2).

*Western blot analysis of rhIGF-1*

**Figure 3:**
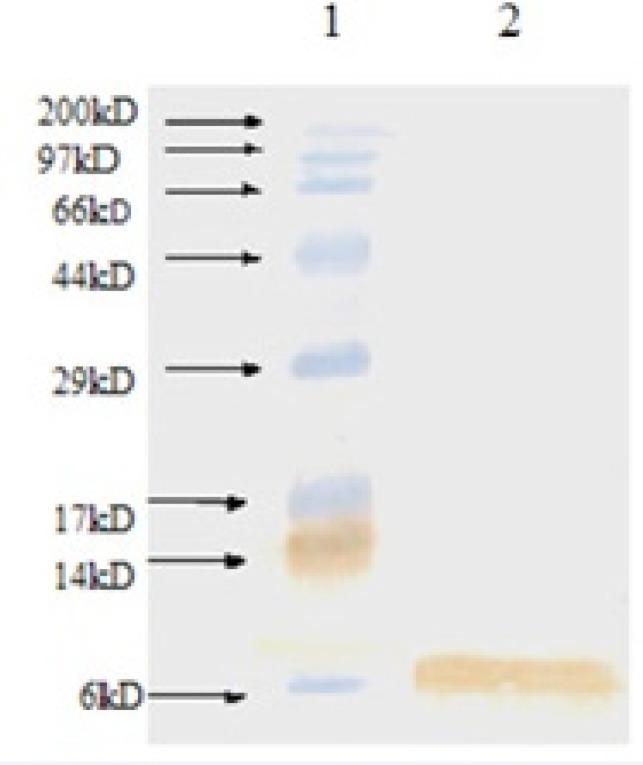
Western blot analysis of produced rhIGF-1 using anti-human IGF-1 antibody. Lane 1: Molecular weight marker, lane 2: produced rhIGF-1 in shaking flask experiment step (0.1 mM IPTG, 32 ^o ^C, TB medium).

The expressed rhIGF-1 was also analyzed by Western blotting using anti-human IGF-1 antibody (Abcam-USA) for further identification. According to Figure 3, Western blotting analysis approved production of rhIGF-1.

*Shaking flasks experiment*

In this study, an L_9_ orthogonal array of the Taguchi method was implemented for three times to investigate the effects of type of medium culture , temperature of induction and amount of inducer on shaking flask (Table 1).Then, the results of experiments (Table 3) were analyzed by ANOVA analysis (Figure 5).

**Table 3:** Type of medium, induction temperature and amount of inducer impact on rh-IGF-1 production.

DCW (g/L)	Final OD_600_	IGF-1 concentration (g/L)	Expression percent (%)	Total protein (g/L)	Process duration (h)	Inducer concentration (mM)	Type of medium	Induction temperature (^o^C)
3.15	6.7	0.03	2	1.575	16	0.05	LB	24
3.15	6.7	0.03	2	1.575	16	0.1	TB	24
3	6.4	0.25	15	1.65	16	0.2	32y	24
2.95	6.3	0.086	5	1.53	14	0.1	LB	28
2.95	6.3	0.157	10	1.57	14	0.2	TB	28
3.24	6.9	0.54	30	1.8	14	0.05	32y	28
3.05	6.5	0.19	12	1.59	14	0.2	LB	32
2.95	6.3	0.283	18	1.57	14	0.1	TB	32
3.3	7	0. 694	35	1.98	14	0.05	32y	32

*Effects of different temperatures on the expression of rhIGF-1 protein*

 Temperature should support cell growth as well as product formation. Also temperature affects plasmid stability and consequently the yield of protein production in culture (24, 25) then temperature as an important factor should be optimized for production process. According to the previous study (24) high temperature (37 ^o^C) is suitable for high dry cell weight (DCW) and high production. Generally, lowering temperature during gene induction lead to improve the quality and folding of the recombinant protein. It has been reported that many of physical and structural properties of inclusion bodies such as size distribution, reversibility and native-like structure depend on cultivation and induction condition (15, 16, 17). Although low growth temperatures results in low growth rate and finally low cell density, but in this study we overcame this problem by choose of suitable amount of another factors base on experimental design and Taguchi method.

**Figure 4:**
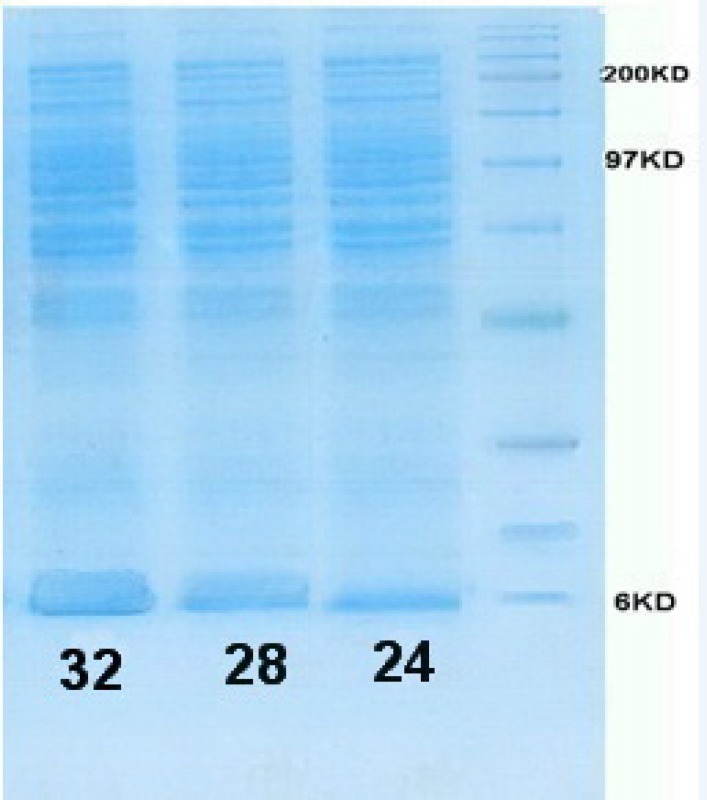
SDS-PAGE gel of rhIGF-1 expression in shaking flasks experiment:

**Figure 5:**
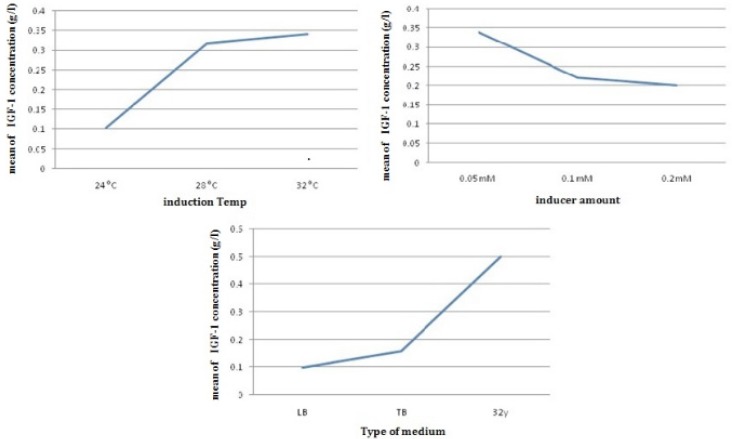
The average effect of induction temperature, inducer amount and type of medium on rhIGF-1 concentration (g/L) in batch cultures of E.oli Origami (B/DE3).

According to L9 orthogonal array, the *E. coli* Origami (B/DE3) /pET15-hIGF-1 expression system was induced at 24, 28 and 32 ^°^C (Table 1). The cells were then harvested for SDS-PAGE analysis to determine the preferable expression temperature. The results in Figure 4, Figure 5 and Table 3 revealed that the highest production titer (0.694 g/L) of protein was achieved at 32 ^o^C and the expression level was dramatically reduced when the culture temperature varied from this critical point. Although the productivity of protein at 28 and 32 °C was near to each other, the fermentation process time at 28 °C was two times longer than that at 32 °C. Thus, the preferable induction temperature was 32 °C.

32y culture medium, temperature of induction 24 ^o^C, 28 ^o^C, 32 ^o^C and 0.05 mM IPTG. (6 hours after induction).

*Effects of different IPTG concentrations on the expression of rhIGF-1 protein*

 The potency of the promoter, the presence or absence of repressor genes on a plasmid, the cellular location of the product, the response of the cell to recombinant protein expression, the solubility of the produced protein and the characteristics of the protein itself affect on inducer amount (17). For the expression vector, pET15-rhIGF-1, the foreign protein expression was triggered by adding IPTG into the culture medium. In other work (26, 27), the expression levels of recombinant proteins changed markedly with IPTG concentrations, showing that the effect of IPTG concentration on recombinant protein production is dependent on the speciﬁc characteristics of the recombinant protein, the host cell and the culture conditions. According to past studies (24), When the IPTG concentration is too low; the foreign protein could not be fully expressed, whereas in this study the most production was observed in a too low amount of inducer concentration. This achievement plays a critical role in industry. Also, the high IPTG concentration would be harmful to cell growth (20). In this work, IPTG concentration was examined from 0.05 to 0.2 mM. As shown in Table 3 and Figure 5, the highest protein productivity was achieved by 0.05 mM IPTG induction.

*Fermentor expe* *riment*

Simultaneously, optimizing glucose concentration and induction time in the selected shake flask condition was performed. Figure 7 shows the SDS-PAGE gel of total cell protein in optimized condition. Over-expression of rh-IGF-1 was conﬁrmed by Western analysis. Blotted membrane shows that the expressed recombinant protein is exactly rh-IGF-1 and it is the same as the standard rh-IGF-1. Quantitative analysis of the collected samples revealed that the expression level of recombinant protein was 40%.

*Effect of Glucose on expression level*

**Figure 6:**
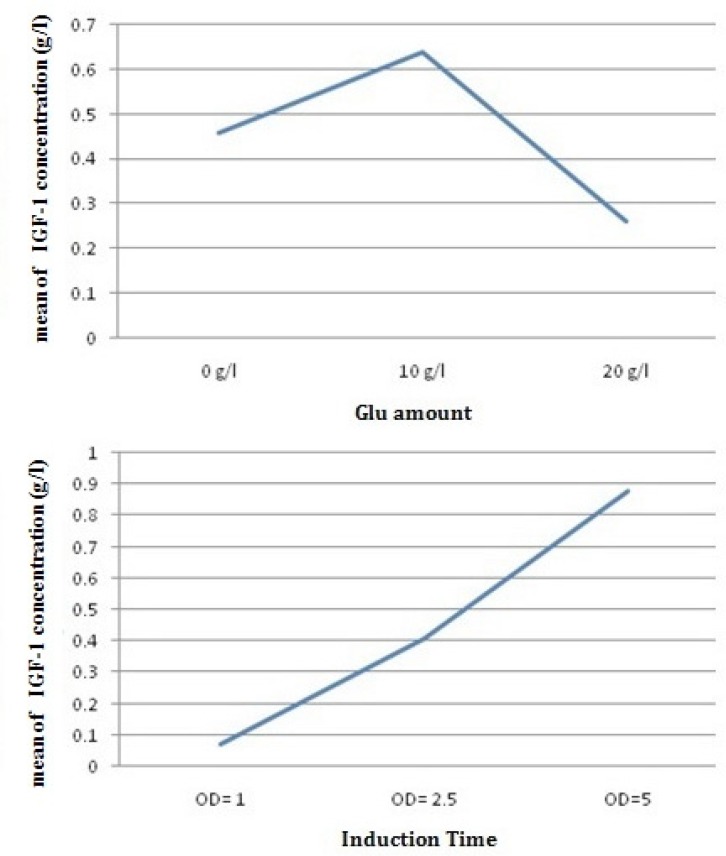
The average effect of glucose amount and induction time on rhIGF-1 concentration (g/L) in batch cultures of *E.coli *Origami (B/DE3).

Glucose is required for cell growth and DCW increasing. But in this study, as a result, initial glucose concentration should be limited because the expression of foreign protein in the presence of glucose with higher concentration in medium (32y) will not be expressed properly. The concentration of cellular cAMP increases when there is not enough glucose in the medium. Accumulated cAMP in the cytoplasm activates the CRP. Active CRP causes the expression of genes of the lac promoter, therefore leading to pre-induction expression. In other words, excess glucose in the medium has an inhibitory effect on cAMP and the CRP–cAMP complex production (25, 27). Hence, the effects of initial glucose concentration on DCW and the speciﬁc growth rate are presented in Table 4, and further details are shown in Figure 6. Further increments of the initial glucose concentration of 10 g/L have an inhibitory effect on growth and cause a reduction in the speciﬁc growth rate. It has been reported that increasing the initial glucose concentration to 40–50 g/L will cause the cessation of growth (27–29). Enhancing the initial glucose concentration up to 10 g/L, increases cell capacity for glucose up-taking. But, when the initial glucose concentration reaches 20 g/L, microorganism is unable to consume the excess glucose in the medium taking extra time for the microorganisms to consume all the glucose in the medium (28, 29). The effect of the initial glucose concentration on of rh-IGF-1 expression, and productivity of rh-IGF-1 in batch culture is presented in Table 4, and more details are given in Figure 6. After induction, concentration and production of rhIGF-1showed a sharp increase that is consistent with previous studies (25, 27, 28, and 29). Increasing the initial glucose concentration from 0 to 10 g/L will increase the concentration of rhIGF-1 from 0.07 to 1.26 g/L. However, when initial glucose concentration increases to 20 g/L, rh-IGF-1, the concentration significantly decreases (0.05 g/L). Considering the productivity and ﬁnal concentration of rh-IGF-1, the value of 10 g/L was chosen as the optimum value of the initial glucose concentration.

**Table 4:** Initial glucose concentration and induction time effect on rh-IGF-1 production

DCW (g/L)	Final OD_600_	IGF-1 concentration (g/L)	Expression percent (%)	Total protein (g/L)	Process duration(h)	Induction Time (OD_600_)	Initial glucose concentration (g/L)
2.86	6.1	0.07	5	1.43	14	1	0
3.43	7.3	0.11	7	1.7	16	1	10
2.8	6	0.02	2	1.4	14	1	20
4.18	8.9	0.41	18	2.29	14	2.5	0
4.27	9.1	0.59	25	2.39	16	2.5	10
3.8	8.1	0.24	12	2	14	2.5	20
4.98	10.6	0.89	32	2.8	18	5	0
5.54	11.8	1.26	38	3.32	18	5	10
4	8.7	0.53	24	2.24	16	5	20

*Effects of different induction times on the expression of rhIGF-1 protein*

 Induction is generally carried out at early or mid-log phase. However, there are reports that induction in late-log phase or even stationary phase (28) can inﬂuence the expression levels. To investigate this effect, the Utrecht group (28, 29) carried out induction at early, mid- and late-log phase, at early stationary phase as well. Overall, induction at early log phase provided the best results. Induction in Stationary phase was counterproductive. In other word, total expression was completely lost for half of the targets and decreased for the rest. Totally, according to the previous investigation, timing of induction may be regarded as a useful parameter to vary optimization of production process (30).

**Figure 7:**
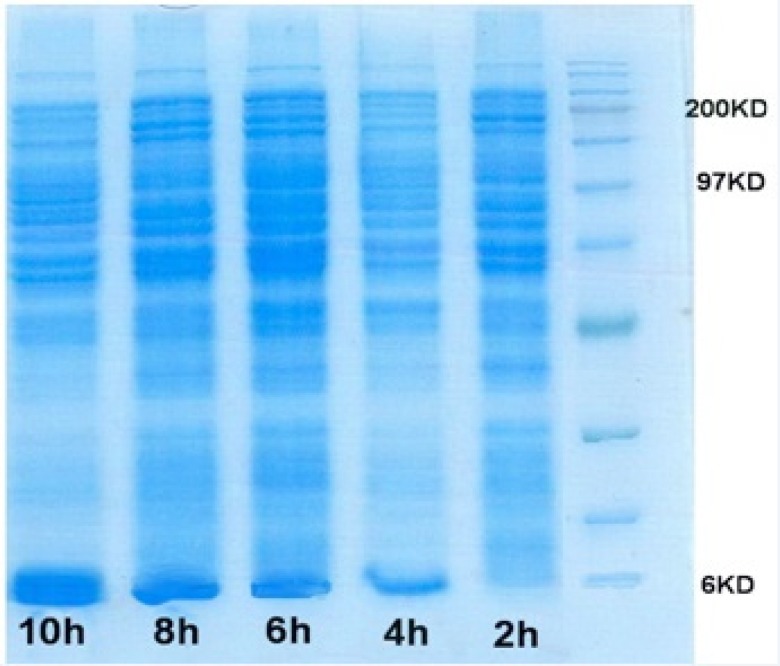
SDS-PAGE gel of rhIGF-1 expression in Fermentor experiment

In this study, to determine the optimal suitable induction timing, the time course proﬁle of cell growth at 32 ^o^C was established and the effects of different induction timing were evaluated by adding 0.05 mM IPTG at different stages of growth phase in 32 y medium. The expression levels were analyzed through SDS-PAGE (Figure 7). At different induction timings, the concentration of rhIGF-1 protein varied in a wide range from 0.07 to 1.26 g/L, the highest value observed when cells were induced at the end of exponential phase (OD _600_ = 5)(Figure 6). This result suggested that induction timing might be a very sensitive factor for the efficient expression of recombinant protein. The optimal induction time was determined through analyzing induced cell samples. The results were shown the expression level of hIGF-1 protein increased up to 1.26 g/mL (Table 4).

at the end of exponential phase (OD600=5) and 10 g/L glucose. (2, 4, 6, 8 and 10 hours after induction).

## Conclusion

The production of rhIGF-1 in the *E. coli* (origami) was dependent on culture media and fermentation conditions. Three different media (LB, TB and 32y medium) were used for batch cultivation of recombinant *E. coli* and production of rh-IGF-1. 32y was shown to be superior in terms of medium simplicity and CDW. Also, it was concluded that induction conditions including induction temperature, amount of inducer and induction time significantly influenced on recombinant protein production in batch culture. Our data showed that the optimal condition was induction by 0.05 mM IPTG at 28 ^o^C at the end of exponential phase. Moreover, this medium was optimized in terms of initial glucose concentration. It was found that initial glucose concentration of 10 g/L will result in the best levels for the ﬁnal concentration of rhIGF-1 and productivity of the process. Totally, the ﬁnal concentration of rhIGF-1 reached to the value of 1.26 g/L that is one of the best reported amounts (31).
